# QUALITY OF LIFE EVALUATION AND ASSOCIATED FACTORSIN ASTHMATIC CHILDREN AND ADOLESCENTS ATTENDED IN A SPECIALIZED OUTPATIENT CLINIC

**DOI:** 10.1590/1984-0462/2020/38/2018172

**Published:** 2020-01-13

**Authors:** Fernanda Chedid de Souza Fontan, Sérgio Wilson Duwe, Karoliny dos Santos, Jane da Silva

**Affiliations:** a Universidade do Sul de Santa Catarina, Palhoça, SC, Brazil.; b Universidade Federal de Santa Catarina, Florianópolis, SC, Brazil.

**Keywords:** Asthma, Quality of life, Child, Adolescent, Asma, Qualidade de vida, Criança, Adolescente

## Abstract

**Objective:**

To evaluate the quality of life and its association with disease control, severity, allergic comorbidities and adherence to treatment in children and adolescents with asthma.

**Methods:**

A cross-sectional study that included children and adolescents aged seven to 17. The Paediatric Asthma Quality of Life Questionnaire (PAQLQ) was used to assess their quality of life. Sociodemographic and clinical data were obtained from the chart and from a questionnaire. Descriptive statistics were performed and chi-square or Fisher’s exact tests were used to verify the existence of associations between quality of life and disease control, severity, comorbidities and adherence to treatment. The level of statistical significance was set at p<0.05.

**Results:**

101 children/adolescents were evaluated (62.4% boys), with a mean age of 10.1 years. On average, the PAQLQ score was ≤5.9 points, indicating moderate / severe quality of life impairment. Higher levels of control, as well as higher disease severity, were associated with higher quality of life impairment, both in total PAQLQ score and domains (p<0.05). The presence of comorbidities was also associated with higher quality of life impairment (p=0.01), except in the emotional function domain. Adherence to treatment showed no association with quality of life.

**Conclusions:**

Children and adolescents with asthma present impairment in their quality of life, and this is related to poorer control and severity of the disease, as well as to the presence of allergic comorbidities.

## RESUMO

**Objetivo:**

Avaliar a qualidade de vida e sua associação com controle da doença, gravidade, comorbidades alérgicas e adesão ao tratamento em crianças e adolescentes com asma.

**Métodos:**

Estudo transversal que incluiu crianças e adolescentes com idade entre sete e 17 anos. O *Paediatric Asthma Quality of Life Questionary* (PAQLQ) foi utilizado para avaliar a qualidade de vida. Dados sociodemográficos e clínicos foram obtidos a partir do prontuário e de um questionário. Foi realizada estatística descritiva e o teste do qui-quadrado ou o teste exato de Fisher foi utilizado para verificar existência de associações entre qualidade de vida e controle da doença, gravidade, comorbidades e adesão ao tratamento. O nível de significância estatística adotado foi de p<0,05.

**Resultados:**

101 adolescentes/crianças foram avaliados (62,4% meninos), com média de idade de 10,1 anos. Em média, a pontuação do PAQLQ foi ≤5,9 pontos, indicando comprometimento moderado/grave da qualidade de vida. Piores níveis de controle e a maior gravidade da doença estiveram associados ao maior comprometimento da qualidade de vida, tanto no escore total do PAQLQ quanto por domínios (p<0,05). A presença de comorbidades também esteve associada ao maior comprometimento da qualidade de vida (p=0,01), exceto no domínio função emocional. A adesão ao tratamento não demonstrou associação com a qualidade de vida.

**Conclusões:**

Crianças e adolescentes com asma apresentam prejuízo na qualidade de vida, e este está relacionado com pior controle e maior gravidade da doença, assim como com a presença de comorbidades alérgicas.

## INTRODUCTION

Asthma is a heterogeneous chronic obstructive disease, with airway inflammation as its main characteristic. The development and maintenance of symptoms result from a complex interaction between specific and genetic factors, in addition to the environmental exposure to allergens. This inflammation is characterized by lower airway hyperresponsiveness and infiltration of inflammatory and structural cells, associated with a variable and reversible limitation of airflow.^1-3^

As a chronic disease, asthma has a high economic impact, being the chronic disease most related to care in emergency departments in the pediatric age range.^4^ According to the Brazilian Technology Department of the public health system (*Departamento de Informática do Sistema Ûnico de Saúde* – DATASUS), asthma was the third cause of hospitalization in the age group 0–19 years in 2016, and the costs of these cases reached R$ 34,551,874.86.^5^ Besides the economic impact, and due to its chronic nature, asthma can also lead to physical and social constraints, which, consequently, can negatively affect the quality of life (QOL) of those who have the disease.

There is a growing consensus that health-related QOL assessments are also necessary to provide a complete overview of the health status of children.^6^ Thus, a health-related QOL assessment administered to asthmatic children through questionnaires should be part of their systematic follow-up.^6^ These questionnaires are important tools because, combined with clinical measures, they can offer a complete evaluation of the impact of the disease and the potential effect of a particular treatment on the general well-being of children.^7^

Clinically, it is necessary to monitor QOL in pediatric asthma over time, since a consistently low QOL is associated with poorly controlled asthma. This knowledge can help in clinical decisions to minimize risks and guide adjustments in pediatric medication, as it can assist in the identification of subgroups of patients with risk of adverse asthmatic outcome.^8^

Despite the worldwide call for QOL investigation as a complementary form of evaluating diseases and patients, few studies have focused on the pediatric asthmatic population. Considering the above, this study aimed to assess the QOL in asthmatic children and adolescents treated in the Municipal Polyclinic of Palhoça – Universidade do Sul de Santa Catarina (Unisul), as well as evaluate associations between QOL and disease control and severity, allergic comorbidities, and treatment adherence.

## METHOD

This is an observational cross-sectional epidemiological study, conducted in the Municipal Polyclinic of Palhoça, linked to the School of Medicine at Unisul. We included children and adolescents aged 7 to 17 years, diagnosed with asthma, who had a medical appointment at the Outpatient Clinic School of Pneumopediatrics and Allergology of the polyclinic mentioned above, from March 2016 to June 2017. The study sampling was non-probabilistic, that is, considered the patients who showed up for their appointment until reaching the size determined by the sample calculation. We excluded patients with other associated respiratory diseases or who presented neurological conditions or cognitive impairment that could prevent them from answering the QOL-related questions.

All parents or guardians, as well as the children and adolescents, were informed about the procedures and signed the informed consent form and the agreement form, respectively. The Human Research Ethics Committee of Unisul (REC-Unisul) approved this study under report number 1,375,380/2015.

The parents or guardians answered the questionnaire during an interview, providing the following information: age, gender, family history of allergic diseases, allergic comorbidities, symptomatology, treatment adherence, environmental exposures, and indicators of socioeconomic status. Information about medications used in the asthma treatment was preferentially collected from medical records, as well as data on weight and height (measured during the medical appointment, on the same day the questionnaire was administered) and pulmonary function tests (when available).

We adopted the criteria described by the Global Initiative for Asthma (GINA)^1^ to classify asthma severity and level of control. Based on answers to four questions related to the prior four weeks (presence of daytime symptoms more than twice a week, nocturnal symptoms, use of reliever medication more than twice a week, and activity limitation), we classified the disease as:

- Well controlled, when none of the questions received a positive response.
- Partly controlled, when one or two questions received a positive response.
- Uncontrolled, when three or four questions received a positive response.

Regarding severity, the disease was classified as:

- Mild, when asthma was well controlled with reliever medication (short-acting bronchodilator) or low-dose inhaled corticosteroids, leukotriene receptor antagonists, or chromones.
- Moderate, when the disease remained well controlled with low doses of inhaled corticosteroids/long-acting bronchodilator.
- Severe, when the patient needed treatment with high doses of inhaled corticosteroids/long-acting bronchodilator to prevent asthma from becoming or remaining “uncontrolled” despite this treatment.^2^

We used the full version of the Paediatric Asthma Quality of Life Questionnaire (PAQLQ),^9^ validated and adapted for Brazilian Portuguese.^10^ PAQLQ consists of 23 questions answered by children or adolescents that cover 3 different domains – symptoms (10 questions), activity limitation (5 questions), and emotional function (8 questions). The questions concern the previous 7 days, and the responses use a 7-point Likert scale, with 1 being “extremely bothered” or “all of the time” and 7 being “not bothered at all” or “none of the time.” All PAQLQ items are answered in the same way, and their means represent the scores (overall and domain-specific). Higher scores represent better QOL.^10^ The use of the questionnaire was previously authorized by the author.

Considering an expected frequency of 38% of controlled and 14% of partly controlled or uncontrolled asthma among individuals with no or mild QOL impairment and prevalence ratio (PR) of 2.7, we estimated that a sample of 100 individuals was sufficient, taking as reference a mild/moderate ratio of 1:1, with a 95% significance level of and an 80% power. The data were entered into Excel and exported to the software Statistical Package for Social Sciences (SPSS), version 16.0. Data were presented as descriptive statistics. We used the chi-square test or Fisher’s exact test to verify the existence of associations between QOL and disease control and severity, comorbidities, and treatment adherence. We also used PR and a 95% confidence interval (95%CI). For the analysis of associations, we classified the overall and domain PAQLQ scores as previously described in the literature.^11^ Therefore, scores ≥6 points indicated no or mild QOL impairment, while scores ≤5.9 points indicated moderate to severe impairment. Also, we chose to dichotomize control (controlled and partly controlled/uncontrolled asthma) and severity (mild and moderate/severe asthma) variables to analyze the associations. The level of statistical significance adopted was p<0.05.

## RESULTS

Between March 2016 and June 2017, 540 patients were treated in the Outpatient Clinic School of Pneumopediatrics and Allergology of the Municipal Polyclinic of Palhoça. Among them, 433 were not eligible for the study because they belonged to a different age group than the one established in the inclusion criteria. Thus, 107 patients were considered eligible for the study. Out of them, we excluded three individuals due to neurological comorbidities that prevented the administration of PAQLQ, resulting in 104 patients included in the study.

The sample comprised 63 boys (62.4%) with a mean age of 10.1±1.9 years. The mean level of maternal schooling was 8.31±4.5 years of complete education. As to the environmental profile, 24 (23.8%) were exposed to smoking at home, 68 (67.3%) had a pet, and 80 (79.2%) lived in an urban area. Regarding the clinical profile, 59 (58.4%) were considered eutrophic, and 35 (34.7%) were obese.

With respect to family history of asthma, 86 participants (85.1%) stated that someone in the family had the disease; among them, 53.5% were the mother, father, and/or siblings of the patient. A total of 99 patients (98.2%) reported having allergic comorbidities, out of them, 95 (94.1%) declared having allergic rhinitis; 46 (45.5%), atopic dermatitis; and 17 (16.8%), some type of food allergy. Almost 91% of the patients were using some kind of asthma medication. A total of 39 patients (38.6%) reported using short-acting beta-2 agonist alone, 14 (13.8%) used inhaled corticosteroid alone, and 38 (37.6%) used a combination of both. Concerning treatment adherence, 85 (93%) parents or guardians declared that the patients used the medication as prescribed by the doctor; 6 (6.6%), that the patients did not use it regularly, claiming forgetfulness; and 5 (5.4%), that the patients did not receive free medication, and they lacked the financial means to buy it.

As to disease control, 43 patients (42.6%) had controlled; 32 (31.7%), partly controlled; and 26 (25.7%), uncontrolled asthma. Relating to the severity, most of the studied sample presented moderate (51.5%) to severe (26.7%) asthma.

**Figure 1:**
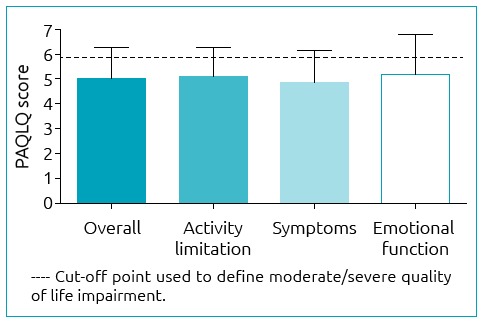
Mean overall and domain Paediatric Asthma Quality of Life Questionnaire scores.

Figure 1 presents the mean overall and domain PAQLQ scores. According to the established cut-off point, the population investigated presented moderate/severe impairment in the overall QOL score (mean=5.0; median=5.3; and standard deviation – SD=1.3) in all domains: “activity limitation” (mean=5.0; median=5; and SD=1.3), “symptoms” (mean=4.8; median=5.1; and SD=1.3), and “emotional function” (mean=5.2; median=5.2; and SD=1.6). Among the domains, 71 patients (70.3%) had moderate/severe QOL impairment in “activity limitation”; 82 (81.2%) in “symptoms”; and 57 (56.4%) in “emotional function”.

As shown in Table 1, asthma control and severity were associated with overall and all domain PAQLQ scores. Patients with moderate to severe QOL impairment had greater chances of having moderate/severe asthma (PR=2.03; 95%CI 1.03–3.93; p=0.04) and were more likely to have the disease partly controlled/uncontrolled (PR=2.39; 95%CI 1.17–4.9; p=0.01).

**Table 1:** Clinical factors – asthma control and severity – related to the type of impairment reported on components of the Paediatric Asthma Quality of Life Questionnaire.

Associated factors	Quality of life impairment		PR	(95%CI)	p-value
	None/mild n (%)	Moderate/severe n (%)			
Asthma control					
Overall PAQLQ					
Controlled asthma	16 (37.2%)	27 (62.8%)	1		
Partly controlled/uncontrolled	09 (15.5%)	49 (84.5%)	2.39	(1.17–4.90)	0.01
PAQLQ – Activity limitation					
Controlled asthma	20 (46.5%)	23 (53.5%)	1		
Partly controlled/uncontrolled	10 (17.2%)	48 (82.8%)	2.7	(1.41–5.16)	0.001
PAQLQ – Symptoms					
Controlled asthma	15 (34.9%)	28 (65.1%)	1		
Partly controlled/uncontrolled	04 (6.9%)	54 (93.1%)	5.05	(1.8–14.2)	<0.001
PAQLQ – Emotional function					
Controlled asthma	30 (69.8%)	13 (30.2%)	1		
Partly controlled/uncontrolled	14 (24.1%)	44 (75.9%)	2.89	(1.76–4.75)	<0.001
Asthma severity					
Overall PAQLQ					
Mild asthma	09 (40.9%)	13 (59.1%)	1		
Moderate/severe asthma	16 (20.3%)	63 (79.7%)	2.02	(1.03–3.93)	0.04
PAQLQ – Activity limitation					
Mild asthma	14 (63.6%)	08 (36.4%)	1		
Moderate/severe asthma	16 (20.3%)	63 (79.7%)	3.14	(1.80–5.40)	<0.001
PAQLQ – Symptoms					
Mild asthma	08 (36.4%)	14 (63.6%)	1		
Moderate/severe asthma	11 (13.9%)	68 (86.1%)	2.61	(1.20–5.70)	0.02
PAQLQ – Emotional function					
Mild asthma	15 (68.2%)	07 (31.8%)	1		
Moderate/severe asthma	29 (36.7%)	50 (63.3%)	1.86	(1.20–2.80)	0.01

PR: prevalence ratio; 95%CI: 95% confidence interval; PAQLQ: Paediatric Asthma Quality of Life Questionnaire.

Table 2 describes the clinical factors – treatment adherence and allergic comorbidities – related to PAQLQ. Allergic comorbidities were associated with moderate/severe QOL impairment (p=0.01), except in the “emotional function” domain (p=0.10). Treatment adherence showed no association with QOL (p=0.95).

**Table 2:** Clinical factors – treatment adherence and allergic comorbidities – related to the type of impairment reported on components of the Paediatric Asthma Quality of Life Questionnaire.

Associated factors	Quality of life impairment		PR	(95%CI)	p-value
	None/mild n (%)	Moderate/severe n (%)			
Treatment adherence					
Overall PAQLQ					
Yes treatment adherence	21 (24.7%)	64 (75.3%)	1		
No treatment adherence	04 (25.2–2.8%)	12 (75%)	1.01	(0.25–2.55)	0.95
PAQLQ – Activity limitation					
Yes treatment adherence	24 (28.2%)	61 (71.8%)	1		
No treatment adherence	06 (37.5%)	10 (62.5%)	1.51	(0.46–4.67)	0.45
PAQLQ – Symptoms					
Yes treatment adherence	16 (18.8%)	69 (81.2%)	1		
No treatment adherence	03 (18.8%)	13 (81.2%)	1.00	(0.27–4.80)	0.97
PAQLQ – Emotional function					
Yes treatment adherence	36 (42.4%)	49 (57.6%)	1		
No treatment adherence	08 (50%)	08 (50%)	1.36	(0.46–3.96)	0.57
Allergic comorbidities					
Overall PAQLQ					
Yes comorbidities	23 (23.2%)	76 (76.8%)			
No comorbidities	2 (100%)	0 (0%)			0.01
PAQLQ – Activity limitation					
Yes comorbidities	28 (28.3%)	71 (71.7%)			
No comorbidities	02 (100%)	0 (0%)			0.02
PAQLQ – Symptoms					
Yes comorbidities	17 (17.2%)	82 (82.8%)			
No comorbidities	02 (100%)	0 (0%)			<0.001
PAQLQ - Emotional function					
Yes comorbidities	42 (42.4%)	57 (57.6%)			
No comorbidities	02 (100%)	0 (0%)			0.10

PR: prevalence ratio; 95%CI: 95% confidence interval; PAQLQ: Paediatric Asthma Quality of Life Questionnaire.

## DISCUSSION

QOL assessment complements the evaluation of clinical symptoms, providing an overview to a better picture of the impact of the disease and treatment on the well-being of children and adolescents.^7^ In the present study, the results revealed that asthmatic children and adolescents treated in the Municipal Polyclinic of Palhoça showed moderate to severe QOL impairment. This finding is similar to that found in other works conducted in Brazilian samples, including the PAQLQ validation study performed by Sarria et al.,^10^ which obtained a mean overall score of 5.1.

In the current study, the “symptoms” domain was the most affected PAQLQ component. The literature provides different findings regarding which PAQLQ domain influences QOL the most. While some studies conducted in Turkey, Brazil, and Poland^6,11,12^ revealed that the “activity limitation” domain has the lowest scores – and, therefore, greater impairment –, studies published in Nigeria, Portugal, and Egypt^13-15^ indicate “symptoms” as the most affected domain. These discrepancies in the findings might be justified by different perceptions of limitation related to cultural aspects, level of physical activity, or inclusion criteria adopted in each study. More sedentary samples might be less affected in the “activity limitation” domain and more in the “symptoms” domain, for example. In addition, samples whose patients have worse levels of asthma control – related to symptoms and use of rescue medication – can show a greater impact in the “symptoms” domain.

Several authors indicate that the level of disease control is one of the main influencing factors of QOL in asthmatic patients.^11,16^ In this study, we found that the lower the disease control, the greater the QOL impairment. Matsunaga et al.^11^ assessed QOL using PAQLQ in a sample with the same age group at a reference outpatient clinic in the city of Campinas, São Paulo, and also verified that QOL was related to the level of control, evaluated by the Asthma Control Test (ACT). A study conducted in Turkey^12^ presented similar results, showing a positive correlation between the ACT score and the overall and domain PAQLQ scores, before and after a drug treatment based on the GINA guidelines.

The association between moderate/severe asthma and worse QOL impairment found in the present study is similar to that of previous studies carried out in Brazil and Sweden.^11,17,18^ A Swedish multicenter study^18^ that analyzed the clinical benefit of assessing QOL in children with severe asthma indicated that this group of patients presented a lower overall PAQLQ score when compared to subjects with controlled asthma. Similarly, Souza et al., when classifying the severity of asthma according to the IV Brazilian Guidelines for Asthma Management, demonstrated that lower PAQLQ scores (which reveal a greater QOL impairment) were only associated with cases of persistent asthma.^17^

In the current study, allergic comorbidities were associated with higher QOL impairment. Nonetheless, these findings should be interpreted with caution, given that only a small number of children/adolescents had no allergic comorbidities. Ayuk et al. showed that patients in this age group with a history of atopy had greater QOL impairment.^13^ In the same way, Ozkaya et al. also found that allergic rhinitis negatively affected the QOL in children.^19^ These findings corroborate the results of the present study, suggesting that patients with allergic comorbidities have a more severe course of asthma due to their atopic nature,^12^ and the severity of the disease is, in turn, related to the impact on QOL. Unlike the studies mentioned above, in this work, the “emotional function” PAQLQ domain was the only one that showed no association with allergic comorbidities. Although we have not assessed the severity of these diseases, they possibly have a lower severity than asthma. Thus, these comorbidities are potentially better tolerated, not involving a significant impact on emotional function.

Despite several publications showing that QOL impairment is lower in children and adolescents who adhere to treatment,^20,21^ this study found no such association, similarly to the findings by Silva et al.^17^ In the present study, the incidence of treatment adherence corresponded to 93%, and was evaluated by questions targeted at parents and/or guardians. Even though we tried to use appropriate questions, the actual adherence could be lower than the one reported, which might have influenced the lack of associations. In addition, we can hypothesize that the adherence declared was mainly related to rescue medication, which only relieves the symptoms. However, as highlighted by Nordlund et al.,^18^ treatment adherence is hard to measure objectively.

The present study has some limitations. Until now, no study attempted to define PAQLQ cut-of points, and, for this reason, we adopted values established according to previous works that categorized the PAQLQ score for statistical analysis. Nevertheless, we chose the PAQLQ to assess QOL because this instrument has been used and validated in several countries^22^ and includes evaluations in both psychological and physical domains specific to the pediatric age group,^16^ in addition to being reproducible and sensitive to changes that are important for the patient.

Despite the worldwide call for QOL investigation as a complementary form of assessing diseases and patients, few studies have focused on younger populations. The QOL assessment is relevant as a measure of health outcome for considering what the individual feels and perceives. Although centered on asthma-related QOL, this study emphasizes the need to prioritize the control of the disease and its comorbidities. In children and adolescents, QOL impairment is not solely associated with asthma. The concomitance of other allergic diseases, particularly allergic rhinitis, worsens the QOL of these children and adolescents, which further reinforces the importance of the concept of a single airway for asthma management.

In conclusion, asthmatic children and adolescents showed moderate to severe QOL impairment, especially due to the symptoms of the disease. In parallel, inadequate control, asthma severity, and allergic comorbidities were associated with a worsening in QOL. Including instruments that evaluate QOL in clinical practice can lead to a deeper knowledge about the impact of asthma, representing an important step toward improving clinical care strategies and assessing the effectiveness of health interventions in this age group.
